# Is it all That Bad When Living with an Intracellular Protozoan? The Role of *Trypanosoma cruzi* Calreticulin in Angiogenesis and Tumor Growth

**DOI:** 10.3389/fonc.2014.00382

**Published:** 2015-01-13

**Authors:** Galia Ramírez-Toloza, Lorena Aguilar-Guzmán, Carolina Valck, Paula Abello, Arturo Ferreira

**Affiliations:** ^1^Faculty of Veterinary Medicine and Livestock Sciences, University of Chile, Santiago, Chile; ^2^Program of Immunology, Institute of Biomedical Sciences (ICBM), Faculty of Medicine, University of Chile, Santiago, Chile

**Keywords:** *Trypanosoma cruzi* calreticulin, angiogenesis, cancer, infectivity, c1q

## Abstract

The immune system protects against disease, but may aberrantly silence immunity against “altered self,” with consequent development of malignancies. Among the components of the endoplasmic reticulum (ER), important in immunity, is calreticulin (CRT) that, in spite of its residence in the ER, can be translocated to the exterior. *Trypanosoma cruzi* is the agent of Chagas disease, one of the most important global neglected infections, affecting several hundred thousand people. The syndrome, mainly digestive and circulatory, affects only one-third of those infected. The anti-tumor effects of the infection are known for several decades, but advances in the identification of responsible *T. cruzi* molecules are scarce. We have shown that *T. cruzi* CRT (TcCRT) better executes the antiangiogenic and anti-tumor effects of mammal CRT and its N-terminus vasostatin. In this regard, recombinant TcCRT (rTcCRT) and/or its N-terminus inhibit angiogenesis *in vitro*, *ex vivo*, and *in vivo*. TcCRT also inhibits the growth of murine adenocarcinomas and melanomas. Finally, rTcCRT fully reproduces the anti-tumor effect of *T. cruzi* infection in mice. Thus, we hypothesize that, the long reported anti-tumor effect of *T. cruzi* infection is mediated at least in part by TcCRT.

## Introduction

The immune system protects against disease. However, abnormally silenced protective immunity against “altered self” may lead to the development of malignancies. As such, cancer represents a prominent example of defective immunological surveillance.

Components of the ER play key roles in the development of protective immunity. Among these components, is calreticulin (CRT) that, in spite of its residence in the endoplasmic reticulum (ER), can be translocated to the extracellular milieu, where it displays immune modulating capacities. Work from several laboratories indicates that CRT is an interesting ER candidate to manipulate anti-cancer immunity.

According to the World Health Organization (WHO), Chagas’ disease is endemic in 21 countries, with about 8 million infected people (1). The disease is considered one of the most important neglected tropical infections worldwide, because it causes 15,000 deaths per year and 0.7 million disability adjusted life-years (2). The impact of this parasite on domestic and wild animals (reservoirs) (3) is unknown.

The disease is endemic in Latin America. However, it has now gone global, affecting several hundred thousand people, mainly South American immigrants, in the USA, Canada, Europe, Oceania, and Asia (4), where transmission is independent of the protozoan. In countries without arthropod vectors, transmission is through blood products (5), organ transplants (1, 5), or congenital (6). Infection can also occur *per os* through parasite-contaminated food (7, 8).

The most frequent treatments for Chagas’ disease have been the administration of Benznidazole or Nifurtimox, with reported efficacy in up to 80% of acute cases after a 60-day course, but with frequent severe side effects and drug resistance (9). Although these drugs reportedly may cure the disease in the acute phase, particularly in children, their efficacy in adults, in the indeterminate or chronic phases, has not been determined.

About 80 years ago, Roskin, Exemplarskaja, and Kliueva, investigators from the former Soviet Union postulated an anti-cancer activity of *Trypanosoma cruzi*, based on a toxic effect of this parasitic infection, or parasite extracts, over different tumors, both in experimental animals and humans (10, 11). More recently, it was described the parasite capacity to infect preferentially tumor cells as compared to normal host cells (12). In general, these data suggest an antagonism between *T. cruzi* infection and tumor growth (12). Herein, we will review the available information with regard to possible molecular mechanisms underlying the anti-tumor effects of *T. cruzi* infection, with emphasis on the experimental rational basis leading to the proposal that the parasite utilizes its calreticulin (TcCRT) to protect its host against neoplastic aggressions. We have provided experimental evidences indicating that TcCRT is an antiangiogenic molecule that inhibits proliferation, migration, and capillary morphogenesis in several *in vitro*, *ex vivo*, and *in vivo* (*in ovum)* assays (13–15). On the other hand, TcCRT inhibits the growth of a mammary adenocarcinoma and a melanoma in different experimental animal models (13–16).

## Is it all That Bad When Living with an Intracellular Protozoan?

The work from the investigators from the former Soviet Union, proposed that *T. cruzi* infection potential as a biotherapy for cancer treatment (17–19), opened possibilities for several research lines. They produced a “cancerolytic toxin” [Kliueva and Roskin (KR) preparation], from *T. cruzi* lysed cells. In humans, affected by a variety of tumors, these “toxins,” reduced tumor growth, pain, local inflammation, and bleeding (18). Controversial results followed and the situation, complicated by World War II and “The Cold War,” interrupted or greatly delayed this work (10). Thus, the mechanism and the molecular component responsible for the biotherapy effect have remained largely unknown.

Of note is the proposal in Science journal in 1948, by the immunologist Theodore S. Hauschka and Margaret Blair Goodwin that tumor-bearing mice, concomitantly infected with the lethal *T. cruzi* strain died within 8–13 days post infection. They observed that weight loss in tumor-bearing infected animals was important, *and that tumor growth was almost completely suppressed*. When, in tumor-bearing animals, the infection was treated, the tumors resumed their usual growth rate, and the hosts died of cancer (20). Thus, presence of the parasites was necessary for tumor inhibition. However, the authors’ view (20) that tumor and parasites compete for nutrients with consequent inhibition of the former does not seem now completely satisfactory given the information emerging during the last few years that we review and discuss below.

Earlier this century, experimental data obtained from rats infected with *T. cruzi* parasites and carcinoma induced by 1,2-dimethylhidrazyne, demonstrated that chronic infection may enhance resistance against tumor growth (21). More recent reports, evaluated the tumor-tropism-parasite capacity to infect host cancer cells rather than normal cells. Normally, the invasiveness (tissue-distribution of parasites of different strains of *T. cruzi*) in mice, primarily demonstrated a parasite tropism toward heart tissues, since 46% (40–65%) of parasites are found in this organ. The liver and kidney contained 3–4 times less parasites and even less was found in the spinal cord. Finally, only 3–4% were found in the brain, spleen, and lymph nodes. However, the presence of a tumor in the host leads to *T. cruzi* redistribution between the tissues: the parasites found in the tumor accounted for 18% in the decrease of heart invasion (now down to 28%) and increased invasiveness of spleen and lymph nodes (12). Nevertheless, a relationship between these findings and tumor development was not addressed in these studies.

More recently, a role for *T. cruzi* infection in controlling tumor growth has been revisited at least in two laboratories, including ours (22, 23). Junqueira et al. (23) reported that the use of a recombinant non-pathogenic *T. cruzi* clone as vector of a testis tumor antigen (NY-ESO-1) is efficient in generating T cell-immune responses and protection against cancer cells, thus delaying tumor development in mice.

Most recently, we have corroborated that *T. cruzi* infection greatly reduces the growth of a mammary adenocarcinoma.

## Calreticulin

Calreticulin, a 46 kDa pleiotropic protein, participating as a chaperone and in calcium homeostasis (24), has been described in different organisms such as humans (25), insects (26, 27), nematodes (28–31), protozoa (32–35), and plants (36).

Calreticulin, mainly residing in the ER of all nucleated cells (37), contributes in different processes such as the control of glycoprotein folding quality and binding to monoglucosylated glycans with high mannose content. CRT is also present in the cytosol, nucleus, secretory granules, on the plasma membrane and also free in the extracellular environment (37). There, CRT modulates the immune response against apoptotic cancer cells (38–42). The mechanisms involved in CRT translocation and release to the extracellular milieu are still unknown (43). CRT also promotes cutaneous wound healing (44–46), cell adhesion (37), nuclear export of some steroid hormone receptors (47–49), and the stability or translation of a variety of RNAs (50–54). CRT reaches the cytosol and nucleus by a C-terminal domain-dependent retrotranslocation, after ER calcium depletion (55).

Calreticulin has a globular N-terminus (N), a proline-rich (P) domain, and an acidic C-terminus (37). An S-domain (aa 160–289), within N and P, binds complement component C1, a “danger signal detection module” that initiates the classical complement activation pathway (56, 57). The primary CRT sequence starts with a signal peptide and ends with a KDEL-ER retention sequence (58). Human CRT (HuCRT) and its N-terminal fragment binds laminin (59), with antiangiogenic properties *in vitro* and *in vivo* (60, 61) and inhibit the growth in several tumor models (62–64). Vasostatin, is a CRT 180 amino acid N-terminal fragment, a potent endogenous inhibitor of angiogenesis and suppressor of tumor growth. Vasostatin inhibits vascular endothelial growth factor (VEGF)-induced endothelial cell proliferation and interactions in matrigel and induces cell apoptosis under limiting oxygen availability (65).

### TcCRT and infectivity

*Trypanosoma cruzi*, may use its CRT, a putative universal apoptosis cell marker (39, 41, 42), in an “apoptotic mimicry” strategy to generate “eat me” signals (i.e., by capturing C1 in the area of flagellum emergence), thus facilitating the invasion of host cells. C1q bridges the parasite molecule with host cell surface receptors (66), most likely CRT known as cC1qR (67). Thus, host C1, upon binding to the trypomastigote surface, also promotes parasite infectivity (68). The parasite molecule responsible for recruiting this complement component has been identified as TcCRT (69, 70). Increased parasite infectivity is paralleled by significant increases in TcCRT mRNA levels during early (cell contact and penetration) infection stages of a VERO cell line. In spite of its lysine–aspartic acid–glutamic acid–leucine (KDEL)-ER retrieval sequence, TcCRT does translocate from the ER to the parasite area of flagellum emergence. An augmented capacity to recruit C1, an important “eat me” signal for phagocytic cells follows, thus leading to increased infectivity (41, 68–72).

The TcCRT–C1q interaction can be decreased by anti-TcCRT F(ab′)_2_ antibody fragments (lacking the C1-binding Fc domains) (73). Indeed, passive immunization of mice with these fragments resulted in important decreases in infectivity and improved clinical parameters (69).

Of particular interest and conceivable consequences in pathology is the possibility that, in *T. cruzi* infected individuals, the parasite molecule may promote autoimmune mechanisms (74).

*Trypanosoma cruzi* CRT also binds complement mannan binding lectin (MBL) and Ficolins (75). Together with C1(q,r,s) they are three complement “danger signal” recognition macromolecular modules. Genetically, structurally, and functionally related, they differ in the nature of the recognized danger signals (22). After binding C1(q,r,s), TcCRT or its S and R central domains inhibit the classical pathway of human complement, in a calcium-independent manner (69, 72, 76). More recently, we have also proposed that l-Ficolin binds TcCRT, thus inhibiting the lectin pathway, a likely alternative or concomitant *T. cruzi* strategy to inhibit the host immune response (75). The roles of MBL and Ficolins in the infectivity process are still under study.

## *Trypanosoma cruzi* Calreticulin, a Molecule with Antiangiogenic and Anti-Tumor Properties

Inhibition of tumor angiogenesis, proposed as a cancer therapy almost 40 years ago (77), is a complex process to form new blood vessels, thus providing the necessary supply of nutrients, oxygen, and ways for waste disposal (78). Antiangiogenesis is currently applicable to a wide variety of tumors, frequently as a supplement to other therapies (79).

Our description of TcCRT provides alternative or concomitant explanations for at least an important part of the anti-tumor effect of this parasite infection. Most likely, TcCRT anti-tumor properties derive from its antiangiogenic properties (13, 73). By direct interaction with endothelial cells, probably through a Scavenger-like receptor, TcCRT acts as a potent angiogenesis inhibitor (13, 14, 71). Antiangiogenic agents may generate a primary stressing challenge to a variety of tumor cells. On the other hand, many tumors have a notorious capacity to produce an array of proangiogenic molecules. Of note are VEGF, the platelet-derived endothelial cell growth factor (PD-ECGF), and the acidic and basic fibroblast growth factors (aFGF and bFGF) (80). Thus, tumor growth and metastasis are indirectly, but importantly promoted by these factors.

Angiogenesis modulators behave differently across species. TcCRT and its N-terminal vasostatin-like domain (N-TcCRT) were studied in mammals, *Homo sapiens sapiens* included (13). Thus, recombinant TcCRT (rTcCRT) and its N-terminal domain inhibit capillary growth *ex vivo* in *Rattus rattus* aortic rings, morphogenesis, proliferation, and chemotaxis in human umbilical cord endothelial cells (HUVECs) (13) and *in ovum* angiogenesis in the *Gallus gallus* chorioallantoid membrane (CAM) assay (14). These are valid correlates of important features of angiogenesis *in vivo*. In most of these assays TcCRT was more effective, in molar terms, than HuCRT (13). Of particular interest is the fact that, in the CAM assay, the antiangiogenic TcCRT effect was fully reverted by polyclonal antibodies against rTcCRT (15). We are currently investigating whether the anti-tumor effect of *T. cruzi* infection is reverted by F(ab′)_2_ anti-TcCRT antibody fragments, derived from these immunoglobulins. In such a case, a formal causal link between externalized TcCRT and the anti-tumor effect of *T. cruzi* infection would be established.

**Figure 1:**
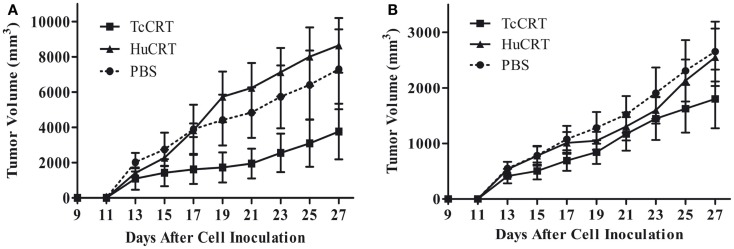
***Trypanosoma cruzi* CRT-mediated tumor growth inhibition**. In both experiments, 5 × 10^5^ murine A/J mammary tumor (TA3 MTXR) cells were inoculated s.c. in A/J female mice, five animals per group. **(A,B)** Together with tumor cells, and every other day, the animals were inoculated s.c. with 50 μg TcCRT or HuCRT or solvent. While TcCRT had a similar anti-tumor effect in both experiments (*p* = 0.0078), HuCRT did not show that effect under these conditions (13). In both experiments, the tumor size was determined with a digital caliper (Mitutoyo Corp., Japan), in a double blind procedure. The formula (*π*/6 × length × width^2^) was used. Data were statistically validated by Wilcoxon Signed Rank test, GraphPad Prism 4. Reproduced with permission from PLoS Neglected Tropical Diseases (13).

In agreement with the previously described facts, inoculation of rTcCRT inhibits by 60–70% the time-course development of a murine mammary metrotexate multiresistant adenocarcinoma (TA3-MTX-R), with a higher efficiency than the human counterpart (13) (Figure 1).

## Concluding Remarks

Recombinant TcCRT, and most likely translocated native TcCRT, mediate mechanisms relevant in the host/parasite interplay: (i) through a central S domain (aa 159–281), it interferes with the earliest stages of the complement activation; (ii) C1, bound to the parasite, promotes infectivity (69); and (iii) through an N-terminal domain (20–193), it interacts directly with endothelial cells and inhibits angiogenesis (13). Thus, prolonged host–parasite interactions may be promoted. Several of these features are variably conserved in the HuCRT, but with lower equimolar efficiency. Thus, when the parasite and human chaperones are compared in equimolar terms, the former displays stronger antiangiogenic effects in *in vitro*, *ex vivo*, and *in vivo* (22, 70) and this is reflected in the compared anti-tumor effects.

Perhaps the TcCRT antiangiogenic effects reflect a parasite evolutionary adaptation to protect its host integrity and, as a necessary consequence, its own (71). Concomitantly, by decreasing angiogenesis, access of immunocompetent cells to the sites of parasite locations may be impaired, as well as subsequent inflammatory consequences, both with possible benefits to the aggressor, although the second strategy could also benefit the host from exaggerated immune reactivity.

The ability of TcCRT to delay solid tumor growth may represent an evolutionary adaptation with consequences in host survival and increased possibilities for the parasite to expand its genome. Based on fundamental Darwinian principles, cancer (i.e., mammary, cervix-uterine, prostate, lung, stomach, among others), taken altogether, have prevalence equivalent to an epidemic. These cancers may have exerted a selective pressure on the parasites, to develop molecular mechanisms to protect their hosts. Our experimental evidences indicate that externalized TcCRT, through its antiangiogenic properties may explain, at least in important part, such mechanisms (70).

**Figure 2:**
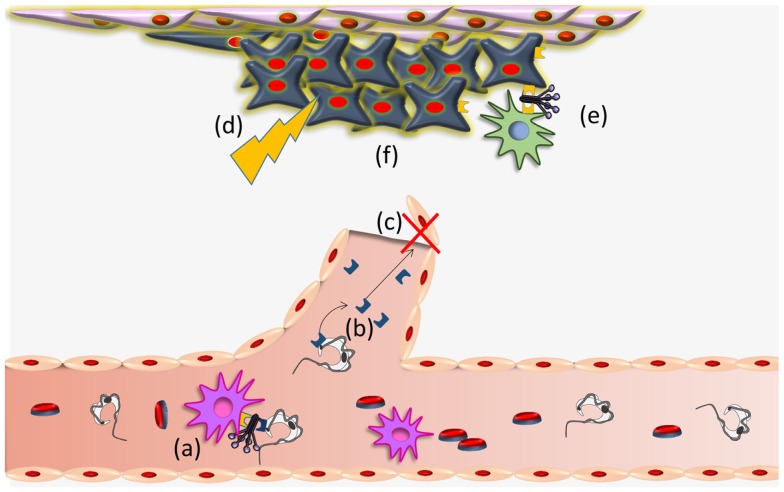
***Trypanosoma cruzi* CRT participates in infectivity and anti-tumor process**. (a) TcCRT, exposed on the parasite surface, binds C1q thus inhibiting the classical pathway of the complement system. TcCRT/C1q interaction participates in the infectivity process binding CRT present on mammalian cells. (b) TcCRT is translocated to the parasite surface and secreted. This TcCRT in the extracellular milieu binds to endothelial cells, (c) inhibiting angiogenesis. (d) This inhibition provokes a stressful environment in the tumor (decreased nutrients and oxygen supply, accumulation of metabolic waste products, etc.) (e) that induces CRT exteriorization on tumor cells. External tumor CRT captures C1, a signal that increases phagocytosis of tumor cells and consequent immunogenicity and (f) reduction of the tumor growth. Whether TcCRT also binds tumor cells *in vivo*, thus promoting tumor immunogenicity, has not been demonstrated.

*Trypanosoma cruzi* CRT-mediated antiangiogenesis, may provoke a stressful environment in the tumor (decreased nutrients and oxygen supply, accumulation of metabolic waste products, etc.) that induce CRT exteriorization on tumor cells. External CRT captures C1, a signal that increases phagocytosis of tumor cells and consequent immunogenicity (16). These possibilities are summarized in Figure 2. Other stressful agents (i.e., UV, anthracyclins) (39–42, 81) also mediate CRT translocation with similar immune stimulating consequences. The possibility that a concomitant immune boost, mediated by other means, is promoted by the infection (23), is also conceivable.

Finally, given the current evidences, the old proposal that tumor and parasites compete for nutrients with consequent inhibition of the former (20), now seems less likely.

## Conflict of Interest Statement

The authors declare that the research was conducted in the absence of any commercial or financial relationships that could be construed as a potential conflict of interest.
